# How Much Does That Treatment Cost?

**Published:** 2013-01-01

**Authors:** Pamela Hallquist Viale

Having been an advanced practitioner (AP) in oncology for many years, I have witnessed the development of several innovative and effective therapies. When I started my career in the early 1980s, we had less than a dozen active antineoplastic agents; within 30 years dozens of new agents have been developed. Other practice changes have included new methods of radiation therapy, techniques to minimize toxicity, bone marrow transplantation, and the development of a whole new way of thinking about treatment: targeted therapies.

These advances have made a great difference in the treatment and extended survival of countless patients with cancer. It has been a privilege to be part of these incredible discoveries and what they mean to these patients. But what is the cost of extended survival? At what point does our health-care system finally say "no" to the use of antineoplastic therapy, especially when the derived benefit to selected patients can be an added 6 months or less of life? Can our current health-care system continue to provide therapies, extensive laboratory testing, and scans to patients in the last months of their lives? Can we continue to absorb the cost of new treatments, particularly when some of them are similar to less expensive versions already approved for care? 

## The Economic Burden of New Therapies

Over the past decade or so, many new therapies have been approved to treat advanced cancers. The therapeutic value of some of these therapies may be limited, and most are noted to be very expensive—many exceed $25,000 a year, a number that may soon seem reasonable given the recent approval of a drug costing over $100,000 a year (Sorenson, 2012; Meropol & Schulman, 2007). Selected therapies may be "second in class" or "third in class," representing new versions of an already approved drug rather than a new chemical entity or an agent with a truly innovative mechanism of action. Can our current health-care system continue to support the use of these new agents and treatments for patients with advanced cancer? 

A recent editorial published in *The New York Times* eloquently questioned the cost of cancer therapies, when Bach, Saltz, and Wittes at Memorial Sloan-Kettering Cancer Center (MSKCC) reported on the decision not to approve an extremely costly new therapy for their patients with cancer (Bach, Saltz, & Wittes, 2012). This agent, ziv-aflibercept (Zaltrap), is similar to an already approved agent widely used in the treatment of colorectal and other cancers: compare ziv-aflibercept at approximately $11,000 a month with bevacizumab (Avastin) at approximately $5,000 a month (Bach, Saltz, & Wittes, 2012). The decision of MSKCC not to prescribe aflibercept is a brave one; large academic centers are frequently the treatment facilities where newer therapies are offered and where patients are often referred. 

The costs of cancer therapy can influence choices in treatment (Neumann, Palmer, Nadler, Fang, & Ubel, 2010). Although national guidelines exist to assist practitioners in evidence-based decision-making regarding therapy for cancer, one survey noted that 84% of oncologists reported that patients’ out-of-pocket spending influenced their recommendations for treatment (Neumann et al., 2010). 

The cost of cancer care has attracted the attention of providers, and APs are undoubtedly in the middle of this issue. Whether we serve on our institutions’ Pharmacy and Therapeutics Committees to determine which new drugs make it to the formulary or struggle with insurance companies to get coverage for our patients’ treatments, the cost of new therapies remains an increasing concern for all APs. The determination of cost vs. benefit needs to be addressed. 

## Addressing the Cost of Cancer Care

The American Society of Clinical Oncology developed a Top Five list to address ways to improve value cancer care (Schnipper et al., 2012). The Top Five recommendations are as follows:

1. For patients with advanced solid tumor cancers who are unlikely to benefit, do not provide unnecessary anticancer therapy, such as chemotherapy, but instead focus on symptom relief and palliative care.
2. Do not use PET, CT, or radionuclide bone scans in the staging of early prostate cancer at low risk for metastasis.
3. Do not use PET, CT, or radionuclide bone scans in the staging of early breast cancer at low risk for metastasis. For individuals who have completed curative breast cancer treatment and have no physical symptoms of cancer recurrence, routine blood tests for biomarkers and advanced imaging tests should not be used to screen for recurrence.
4. Avoid administering colony-stimulating factors to patients undergoing chemotherapy who have a < 20% risk for febrile neutropenia.

This list is a step toward answering some of the questions regarding the cost of therapy, but we clearly need to continue this discussion. The physicians at MSKCC raised important questions regarding the cost of new therapies; in an extraordinary reversal, the manufacturer of ziv-aflibercept decided to reduce the cost of its agent by 50% through offering discounts vs. a true cost in the acquisition of the drug (Pollack, 2012). Sanofi released a statement acknowledging the market resistance to the perceived relative price of the agent, particularly with the low recognition of this new drug.

Health-care costs in general are rising. And although we all want the very best care for our patients with cancer, questions regarding cost vs. benefit must be addressed. Advanced practitioners need to be at the table when cost discussions take place.
